# Supplementary material for: Patterns of Intron Gain and Loss in Fungi
Source: PLoS Biol. 2004 Nov 30;2(12):e422. doi: 10.1371/journal.pbio.0020422 (PMC532390; doi:10.1371/journal.pbio.0020422)
Supplement: Table S1 — Also available at http://genes.mit.edu/NielsenEtAl/. (4.3 MB ZIP). [file pbio.0020422.st001.zip › NielsenEtAl/html/731.html]

AN9069.1.NCU00911.1.MG01191.1.FG00702.1


```
 CLUSTAL W (1.82) Multiple Sequence Alignments - Introns Inserted


Sequence 1: NCU00911.1	405 aa
Sequence 2: FG00702.1	526 aa
Sequence 3: MG01191.1	483 aa
Sequence 4: AN9069.1	441 aa
Alignment Length: 533 aa
Number Identitical Residues: 131 aa
Alignment Score (without introns) 8321


MG01191.1 	----MATPLQIMPLPVWPITFLEDAVVYLSALFTPWFTAFCVLW2LHRYVRLIVHCYSHW
NCU00911.1	--------------------------------------------~---------------
FG00702.1 	MSESEHQNVGRSPLGFAGPLSLSMPSFDFWYSSTFWLYLFLGLW2FHRYFRLLVHCVSHW
AN9069.1  	-------------------------------MHITYMRAFIALF2VYRYLRLVVNLISFY
          	                                                         :  

MG01191.1 	TYKSKPIPSKPSYTSDDVTVVIPTIHDNFDELRPSLESILATKPHELIMVTTADKFEDLQ
NCU00911.1	--------------------MAATLRKP------------ACDHP---------------
FG00702.1 	TYKSKPIPSKPTFTSKDVTVVIPTIHNAFEELRPSLESILACEPAELILVTTHDKRKELQ
AN9069.1  	TFKPIPPPEDSRLTSSDVTVIIPTLDGCGEELERTIETILTNKPHELLLVTIEANKKKAE
          	: .. . ....  ::.. : : .*:    ..   : .:  : .  .    :   . .. .

MG01191.1 	RVAKTL--SSPNIRIFCTQYANKRIQVCEALPK--ITTRITIMADDDVTWPSTMMPWILA
NCU00911.1	----------IKIEVLHVDKANKRLQVCKALEDNHVETEITVMADDDVEWPSTLMPWLLA
FG00702.1 	KLADSL--VFPKVRVLDTPIANKRLQVCEALPK--VETPITIMADDDVTWPSTLMPWILA
AN9069.1  	EMLSRMPAAKSRIRLFTVTHPNKRRQMTRAIPE--VTTTITVFADDDVIWPPKVLTWMLA
          	   .  .:   .:.:: .  .*** *: .*: .  : * **::***** **..::.*:**

MG01191.1 	PFE-DPKIGGVGTCQRVKRVREGGLGLRIWNWLGAAYIERRNFEISATHNMDGGTSCMSG
NCU00911.1	PFE-DDQIGGVGTCQRVKR-IDGDLATRIFNWLGAAYIERRNFEISATHNIDGGTSCMSG
FG00702.1 	PFE-DPEIGGVGTCQRVRREHDGSWSTKAWNWLGAAYIERRNFEISATHNIDGGTSCMSG
AN9069.1  	PFEKDDRYGGVVTCQRLKRATSPTLSQRVWGFLGALYLERRNFDCAATTHIDGGLPCMSG
          	***.* . *** ****::*  .   . : :.:*** *:*****: :** ::*** .****

MG01191.1 	RTGAYRSEILRDYEFLEGFMKEEWW--GKILKADDDNFVSRWLVSHKWKTWIQYEQECEL
NCU00911.1	RTGAYRSRILKGYPFLSGFKSEKWG--KYILNADDDNYVTRWLVAHQWKTWIQYEPECEI
FG00702.1 	RTGAYRSEILSSHDFLHGFKNEKWR--KWILNADDDNFVTRWLVSHQWKTWIQYEKECEI
AN9069.1  	RTVAYKTSILQDDAFTYAFTNEGWWFGKYQLNADDDNFITRWMVSHGWETFMQYHPEAEV
          	** **:: ** .  *  .* .* *  .   *:*****:::**:*:* *:*::**. *.*:

MG01191.1 	ETTLEDNIKFLYQCSRWARSNWRSNWTSLVKERHVWK2QQWWCTYALHIATFTSLAFVFD
NCU00911.1	ETTLETSVKFLYQCSRWARSNWRSNWTSLVHERHVLT2QQPWCTYALHIATFTSLAFVVD
FG00702.1 	ETTLENSTKFLYQCSRWARSNWRSNWTSMVTERYIWK2QQLWCTYALHFATFTSLAFLID
AN9069.1  	QTTLEDNPTFLKQCTRWSRSNWRSNLTSMFDEGHIWH2RQPWSAYAVHMTTLSPPALLGD
          	:**** . .** **:**:******* **:. * ::   :* *.:**:*::*::. *:: *

MG01191.1 	FLILAALWWGTEGWEPVNRNRAIYAQLAFL-AFSKVV~KLVGLFRRHPADIMFLPVSIIF
NCU00911.1	PLLLCSCWWATEGWHANDRYTLLAAEIVFMFGFTKVV~KLVGLFRKNPRDLIYLPVSILF
FG00702.1 	PLLLASCWWGTADWDMQNRRYAFWSQFVFMFAFTKVV~KLMGLFIRNPTDVMFLPVSVVF
AN9069.1  	LGLIFLCHKATASWDEH----AHMLAMSFLAAWMFVI1DWV-----------FLPVSILF
          	  ::     .* .*.           : *: .:  *: . :           :****::*

MG01191.1 	GYFHGLIKIYAGLTLNM0TSWGSRTDGDTDDAHRLAPGPVRCSSLNTP------------
NCU00911.1	GYFHGLIKLYALFTLKH0TSWGSRADGDEHNTFRLQEKPPRSQTMLTPSGPG-LDDLSEK
FG00702.1 	GYFHGLVKLYALITLNM0TSWGSRADGDANDEQRLAPAPQPSIVLKTPPGKGSLIRYNAR
AN9069.1  	GYFHGAIKMYAVMTLSA0TTWGSREGADDFDEERMKRRP-DADPLKKP------------
          	***** :*:** :**.  *:**** ..*  :  *:   *  .  : .*            

MG01191.1 	--RSEHKLPHYMQERDEIVNEKQQMREE-EWEHL-----------------------
NCU00911.1	PLRGSRKTGGVTQSRR-LTTTGTAVSHN-TCEHTDGGTAAVPTDLSPPSLQG-----
FG00702.1 	QKGRQTQAQQVSWEKSDYASYDSSTSYVPIRVHTPMATTPIDTKLYTNESSNTCWAI
AN9069.1  	-YYHKFRLNANDFHHRLVPSVTSADIS------------------------------
          	    . :       :    .
```
